# Deletion of *Kif5c* Does Not Alter Prion Disease Tempo or Spread in Mouse Brain

**DOI:** 10.3390/v13071391

**Published:** 2021-07-17

**Authors:** Brent Race, Katie Williams, Chase Baune, James F. Striebel, Clayton W. Winkler, James A. Carroll, Sandra E. Encalada, Bruce Chesebro

**Affiliations:** 1 Laboratory of Persistent Viral Diseases, Rocky Mountain Laboratories, National Institute of Allergy and Infectious Diseases, National Institutes of Health, 903 South Fourth Street, Hamilton, MT 59840, USA; katie.williams@nih.gov (K.W.); chase.baune@nih.gov (C.B.); striebelj@niaid.nih.gov (J.F.S.); clayton.winkler@nih.gov (C.W.W.); carrollja2@niaid.nih.gov (J.A.C.); bchesebro@niaid.nih.gov (B.C.); 2 Department of Molecular Medicine, The Scripps Research Institute, La Jolla, CA 92037, USA; encalada@scripps.edu; 3 Dorris Neuroscience Center, The Scripps Research Institute, La Jolla, CA 92037, USA

**Keywords:** scrapie, prion, PrP, PrPC, PrPSc, kinesin, KIF5C

## Abstract

In prion diseases, the spread of infectious prions (PrPSc) is thought to occur within nerves and across synapses of the central nervous system (CNS). However, the mechanisms by which PrPSc moves within axons and across nerve synapses remain undetermined. Molecular motors, including kinesins and dyneins, transport many types of intracellular cargo. Kinesin-1C (KIF5C) has been shown to transport vesicles carrying the normal prion protein (PrPC) within axons, but whether KIF5C is involved in PrPSc axonal transport is unknown. The current study tested whether stereotactic inoculation in the striatum of KIF5C knock-out mice (*Kif5c^−/−^*) with 0.5 µL volumes of mouse-adapted scrapie strains 22 L or ME7 would result in an altered rate of prion spreading and/or disease timing. Groups of mice injected with each strain were euthanized at either pre-clinical time points or following the development of prion disease. Immunohistochemistry for PrP was performed on brain sections and PrPSc distribution and tempo of spread were compared between mouse strains. In these experiments, no differences in PrPSc spread, distribution or survival times were observed between C57BL/6 and *Kif5c^−/−^* mice.

## 1. Introduction

Prion diseases are a group of progressive neurodegenerative diseases that occur in humans and animals [1]. Essential to the progression of prion diseases is the conversion of the normal cellular prion protein (PrPC) into a mis-folded, disease-associated conformation known as PrPSc that is usually partially protease-resistant [2]. Accumulation of PrPSc in the central nervous system (CNS) is often observed concurrently with neurode generation, and severe gliosis in the brain [2]. Previous studies have demonstrated that PrPSc can infect neurons, and spread throughout neuronal axons, dendrites and across neuronal synapses [3,4,5,6,7,8,9,10,11,12]. However, the mechanisms by which PrPSc spreads within axons and across synapses remain unclear, and this knowledge is critical for a more complete understanding of prion disease pathogenesis and may provide additional targets for therapies directed against prion diseases.

Transport of PrPC is better understood. Anterograde transport of PrPC by kinesin motors and retrograde transport of PrPC by dynein motors have been reported by several groups in both peripheral nerves and nerves of the CNS [13,14,15,16]. Moreover, using genetics as well as live imaging high-resolution light microscopy approaches, Encalada et al. tracked the transport of individual PrPC vesicles in axons of cultured mouse hippocampal neurons, and identified the kinesin-1C (KIF5C) and dynein heavy chain 1 (DHC1) as the microtubule-based molecular motors drive anterograde and retrograde movement of PrPC vesicles, respectively [17]. In a more recent paper, Heisler et al. identified muskelin as a core component that regulates directionality of the PrPC transport associated with dynein and KIF5C [18]. In the absence of muskelin, PrPC degradation by lysosomes was reduced and PrPC transport to the plasma membrane in exosomes was increased. Although PrPC and PrPSc differ in conformation, it seems plausible to postulate that vesicles carrying PrPSc may also utilize microtubule-based transport mechanisms.

In the current study we tested the role of KIF5C in the tempo of prion disease pathogenesis. Knock-out mice depleted of KIF5C (*Kif5c^−/−^*) and C57BL/6 (B6) genetic controls were inoculated stereotactically with a small volume (0.5 µL) of either 22 L or ME7, two different mouse-adapted scrapie strains with different cellular tropisms [19]. Stereotactic delivery of a small volume of inoculum provided a reproducible method to create a consistent initiation point of infection, without artificial spread due to volume overload at the point of injection. Following inoculation, we euthanized groups of B6 and *Kif5c^−/−^* mice at several pre-clinical time points for immunohistochemical (IHC) analysis or following development of advanced prion disease for a survival curve analysis. Early, pre-clinical times were chosen to be able to track new PrPSc spread from the point of infection to distant locations. IHC directed against PrP was used to detect PrPSc in formalin-fixed brain tissue. Multiple matched regions from each brain were examined and PrPSc distribution and spreading kinetics were compared between strains. No differences in PrPSc spreading kinetics, distribution of PrPSc or survival times were observed between C57BL/6 and *Kif5c^−/−^* mice suggesting PrPSc does not spread anterogradely in the CNS using the KIF5C kinesin isoform.

## 2. Materials and Methods

### 2.1. Mice

All mice were housed at the Rocky Mountain Laboratory (RML) in an AAALAC accredited facility in compliance with guidelines provided by the Guide for the Care and Use of Laboratory Animals (Institute for Laboratory Animal Research Council). Experimentation followed RML Animal Care and Use Committee approved protocol # 2015-060-E. The generation of KIF5C knock-out mice (referred to as *Kif5c^−/−^* in this manuscript) has been described previously [17]. Deletion of the *Kif5c* gene from the mice used in this study was confirmed using routine PCR methodology. C57BL/6J (#000664) (B6 in this manuscript) controls were obtained from The Jackson Laboratory.

### 2.2. Stereotactic Surgery and Microinjection of 22 L and ME7 Prions

Targeted microinjection of the striatum was performed on all the mice used in both the kinetic experiments and survival curve study. Age-matched, young adult mice were anesthetized with isoflurane and prepared for surgery by shaving the hair from the dorsal surface of the skull and applying chlorhexidine-based surgical scrub (BD Biosciences) to the area. Mice were then positioned on a stereotaxic frame (David-Kopf Instruments) and maintained on isoflurane anesthesia. Using an aseptic technique, a 1 cm midline incision was made in the skin over the dorsal surface of the skull, and the skull was exposed to allow positioning of the drill over the bregma point of reference. From bregma, the coordinates used were +1 mm anteroposterior, +1.7 mm lateral, and −3 mm ventral to the skull surface. These coordinates were selected to target the center of the left striatum and avoid midline vasculature and passing through any ventricles. A small hole was drilled in the surface of the skull prior to placement of the 33-gauge delivery needle and Nanofil syringe (World Precision Instruments, Sarasota, FL, USA). Ten percent prion-infected brain homogenate stocks were injected into the striatum at a rate of 0.25 µL/min with a total of 0.5 µL per mouse (UltraMicroPump III with a Micro4 pump controller; World Precision Instruments). Mice infected with strain 22 L received 1.0 × 10^5^ LD_50s_ and mice infected with strain ME7 received 8.3 × 10^3^ LD_50s_. The needle was kept in place for 2 min following injection to minimize reflux. The skin incision was closed with 5-0 absorbable PDS II suture. Mice were recovered in heated cages after surgery and received a single subcutaneous injection of 0.2 mg/kg buprenorphine for postoperative pain management. Patency of the needles was verified prior to and after all injections.

### 2.3. Kinetic Experiments

Following inoculation, groups of 22 L-infected mice were euthanized at pre-clinical time points of 7, 25, 40 and 60 days post-inoculation (dpi) for histopathologic analysis. ME7-infected mice were all euthanized at 40 dpi. Following euthanasia, brains were removed and immediately placed in formalin and prepared for histology. Group sizes for each time point are provided in Table 1.

### 2.4. Survival Curve

To compare survival times between *Kif5c^−/−^* and B6 mice, we used stereotactic equipment to intracerebrally inoculate 12 female mice per mouse strain with 22 L scrapie (inoculation described above). Mice were observed 3–5 times per week by personnel blinded to mouse genotype for the onset of clinical signs. Once signs were apparent, mice were observed 5–7 times per week and euthanized when advanced signs of disease were present including marked weight loss, ataxia, kyphosis, unkempt appearance, and severe somnolence. A conscious effort was made to euthanize all the mice at an equivalent stage of disease. Statistical analysis of the survival times was performed using GraphPad PRISM software.

### 2.5. Hematoxylin and Eosin (H&E) and Immunohistochemical (IHC) Staining

For each experimental group, 2–5 half-brains were removed and placed in 10% neutral buffered formalin for 3 to 5 days. Tissues were then processed by dehydration and embedding in paraffin. Sections were cut using a standard Leica microtome, placed on positively charged glass slides, and air-dried overnight at room temperature. The following day, slides were heated in an oven at 60 °C for 20–30 min.

For all the 22 L and ME7-infected mice euthanized for pre-clinical time-points, brains were collected and embedded as coronal sections into two different blocks, A and B. Block A contained the rostral part of the brain, including the striatum and needle track. Serial 5 micron sections were cut through block A and every 9th section was stained with a standard protocol of hematoxylin and eosin (H&E) to facilitate localization of the needle track and for observation of the overall pathology. Sections adjacent to the H&E slides containing needle tracks were identified and used for anti-PrP IHC (see below). Block B contained the remainder of the brain and was cut into three pieces that were embedded together. Efforts were made to confirm that the three sections of brain were as uniform as possible regarding location in the coronal plane to fairly score the thalamus, midbrain and rostral pons for PrPSc deposition in the preclinical time point experiments. For all 22 L infected mice, two sections from each coronal face were viewed. For the ME7 infected mice, four sections from each coronal face were viewed from each mouse.

For the survival curve experiment, histology was performed on five mice per strain. Brains were removed, bisected at the sagittal midline and right hemispheres were formalin fixed and embedded for sagittal sectioning. Two sections were viewed from each mouse, for each staining technique (see below).

For all IHC, de-paraffinization, antigen retrieval and staining were performed using the Discovery XT Staining Module. For anti-PrP staining we used monoclonal antibody D13 produced at RML at a dilution of 1:100 [20,21]. Antigen retrieval was achieved using extended cell conditioning with CC1 buffer (Ventana) containing tris-borate-EDTA, pH 8.0 for 100 min at 95 °C as previously described [22]. D13 was diluted in antibody dilution buffer (Ventana) and applied for 2 h at 37 °C. The secondary antibody, biotinylated goat anti-human IgG (Jackson ImmunoResearch, West Grove, PA, USA) was diluted 1:250 in Ventana antibody dilution buffer and applied for 32 min at 37 °C.

For detection of microglia and astroglia, the Discovery XT staining system (Ventana Medical Systems) was also used. To stain microglia, anti-Iba1 antiserum was generated by immunization of rabbits with a 14 amino acid peptide from the C-terminus of the Iba1 protein as previously described [23] and was a generous gift from Dr. John Portis. For detection of astroglia, a polyclonal rabbit anti-GFAP antibody (DAKO Cytomation) was used. For Iba1, antigen retrieval was undertaken using the standard CC1 protocol (cell conditioning buffer containing tris-borate-EDTA, pH 8.0, ~ 44 min at 100 °C). Anti-Iba1 was used at a 1:2000 dilution and applied for 40 min at 37 °C. The secondary antibody was biotinylated goat anti-rabbit IgG (Biogenex Ready-to-use Super Sensitive Rabbit Link) and was applied for 40 min @ 37 °C. For GFAP staining antigen, retrieval was done using the mild CC1 protocol (cell conditioning buffer containing tris-borate-EDTA, pH 8.0, ~12 min at 100 °C). The anti-GFAP antibody was used at a dilution of 1:3500 in antibody dilution buffer, applied for 16 min at 37 °C. The secondary antibody was the biotinylated goat anti-rabbit IgG described above and was applied for 16 min at 37 °C. Detection for both GFAP and Iba1 used a RedMap detection kit and hematoxylin counterstain. Slides for all H&E- and IHC-stained tissues were scanned and photographed using Aperio eSlide Manager and Imagescope software (Leica).

### 2.6. Prion IHC Scoring and Statistical Analysis

Coronal brain sections from the early time point experiments were analyzed for the presence and intensity of PrPSc staining. Four regions of the brain were analyzed; the striatum that included the needle track (nt) and point of inoculation, the thalamus at a location approximately 3.2 mm caudal to the nt, the midbrain section containing the substantia nigra at approximately 5 mm from the nt, and the rostral pons containing the locus coeruleus about 7 mm caudal to the nt. The following intensity scores were given: 0, no PrPSc; 1, weak positive, subtle deposition, only 1–10 focal deposits per section; 2, positive >10 deposits; 3, moderate positivity, patchy confluent distribution; 4, strong positive, nearly confluent PrPSc within the specific brain region. Any brain region scoring ≥1 was considered positive for PrPSc. When the ratio of positive and negative mice differed between mouse strains at a specific time point and brain region, *p*-values were calculated using Fisher’s exact test and are provided in Table 1.

## 3. Results

### 3.1. The 22 L PrPSc Spreading Kinetics in B6 and Kif5c^−/−^ Mice

To determine whether absence of the KIF5C motor would alter PrPSc spread in the mouse brain, we compared spreading rates between *Kif5c^−/−^* and B6 mice. Mice were inoculated stereotactically in the striatum with a 0.5 µL volume of 22 L prion infected brain homogenate. Following inoculation, mice were euthanized at 7, 25, 40 and 60 dpi to detect new PrPSc deposition and monitor their spread. The time points selected cover early stages of the disease when PrPSc deposition and spread was minimal but increasing steadily, providing optimal times to observe differences between the experimental mice.

**Figure 1:**
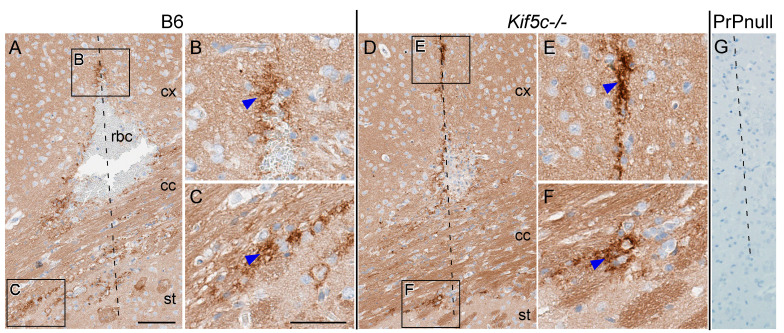
PrPSc staining of 22 L scrapie 7 days post-inoculation. IHC using anti-PrP antibody D13 was performed on coronal sections containing the needle track (nt) from B6 mice (left) and *Kif5c^−/−^* mice (middle) and PrP null mice (right). (**A**,**D**) Overviews of the cerebral cortex (cx), corpus callosum (cc) and dorsal aspect of the striatum (st). The nt is shown with a dashed line, red blood cells (rbc) can be seen in A from hemorrhage post inoculation. (**B**,**C**,**E**,**F**) Higher magnification of the cx, cc and st showing PrPSc closely associated with the nt (blue arrowheads). Similar staining was observed in B6 and *Kif5c^−/−^* mice. (**G**) No cellular prion protein (PrPC) staining is present with D13 IHC on PrP null mouse brain tissue, 7 days post-inoculation. The scale bar in A is 100 µm and applies to panels A,D and G, the bar in C is 50 µm and applies to panels B, C, E and F.

**Figure 2:**
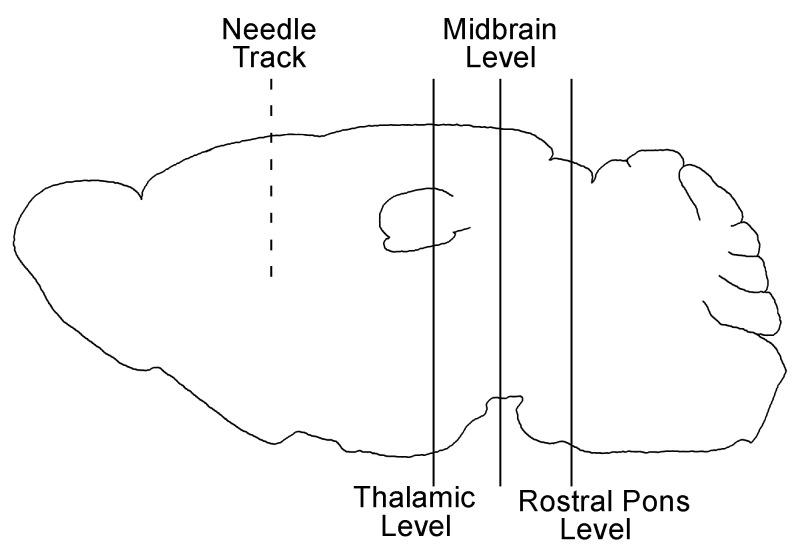
Schematic of the sagittal brain depicting inoculation and coronal section locations. The approximate location of the nt in the striatum (dotted line) and the levels of the three coronal sections (solid lines) analyzed for PrP deposition are shown. The midbrain sections included the substantia nigra and the rostral pons sections included the locus coeruleus.

We first looked for differences in spread at 7 dpi to determine if initial clearance and spread from the point of inoculation differed between B6 and *Kif5c^−/−^* mice. Brains were stained with H&E to facilitate location of the needle track and point of inoculation. Once relevant needle track sections were identified, we performed IHC using monoclonal anti-PrP antibody D13. In both B6 and *Kif5c^−/−^* mice, PrPSc was detected primarily in areas immediately adjacent to the needle track (nt) (Figure 1). Newly formed PrPSc was observed up to 300 µm from the nt in both B6 and *Kif5c^−/−^* mice. Concluding these PrPSc deposits were new was based on experience with historical studies using numerous controls, including normal brain homogenate inoculation and PrP null mice [24]. Pericellular and perivascular PrPSc accumulation was also similar between the two strains of mice at the interface between the corpus callosum and striatum and in focal areas of the striatum near the nt tip (Figure 1). Three other coronal sections at locations distant from the striatum were also analyzed. These three regions, at increasing distances from the nt, included coronal sections to capture the thalamus, midbrain including the substantia nigra and rostral pons, which are regions known for early PrPSc deposition (Figure 2). At 7 dpi, PrPSc accumulation was not observed at any of these locations in either B6 or in *Kif5c^−/−^* mice (Table 1). This finding was not surprising based on previous work following early prion spread [24]. This suggested that screening these more distal areas at later times could indicate differences in spreading kinetics between *Kif5c^−/−^* and B6 mice.

**Figure 3:**
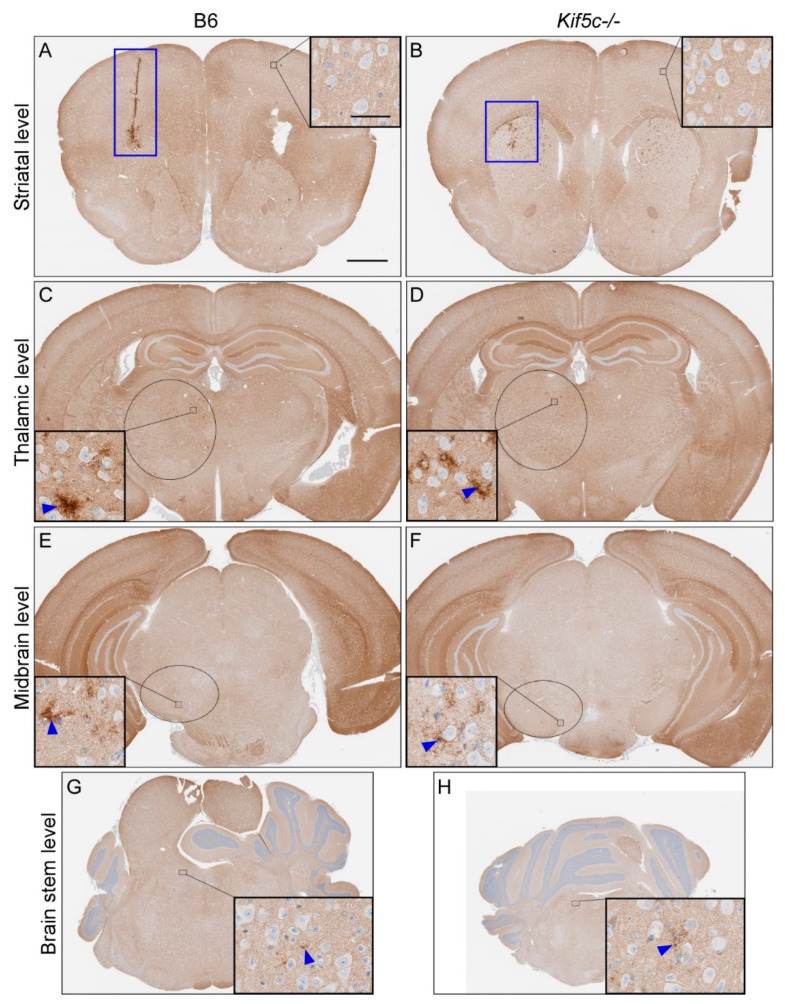
PrPSc staining of mouse brains 25 days post 22 L scrapie inoculation. IHC using anti-PrP antibody D13 was performed on coronal sections from four areas. A representative B6 mouse is shown on the left side, and a *Kif5c^−/−^* mouse on the right. (**A**,**B**) Coronal sections including the striatum. The nt is depicted with a blue box. The insets show higher magnification of the cerebral cortex with normal PrPC staining. (**C**,**D**) Coronal sections through the thalamus. The black oval depicts the approximate borders of the thalamus and the high magnification insets show focal PrPSc staining (blue arrowheads). (**E**,**F**) Midbrain sections including the substantia nigra (black ovals). Insets show higher magnification and PrPSc present in the substantia nigra. (**G**,**H**) Rostral pons sections and higher magnification insets showing subtle PrPSc. The scale bar in A is 1 mm and applies to all the large panels. The scale bar in the inset within panel A is 50 µm and applies to all the insets.

**Figure 4:**
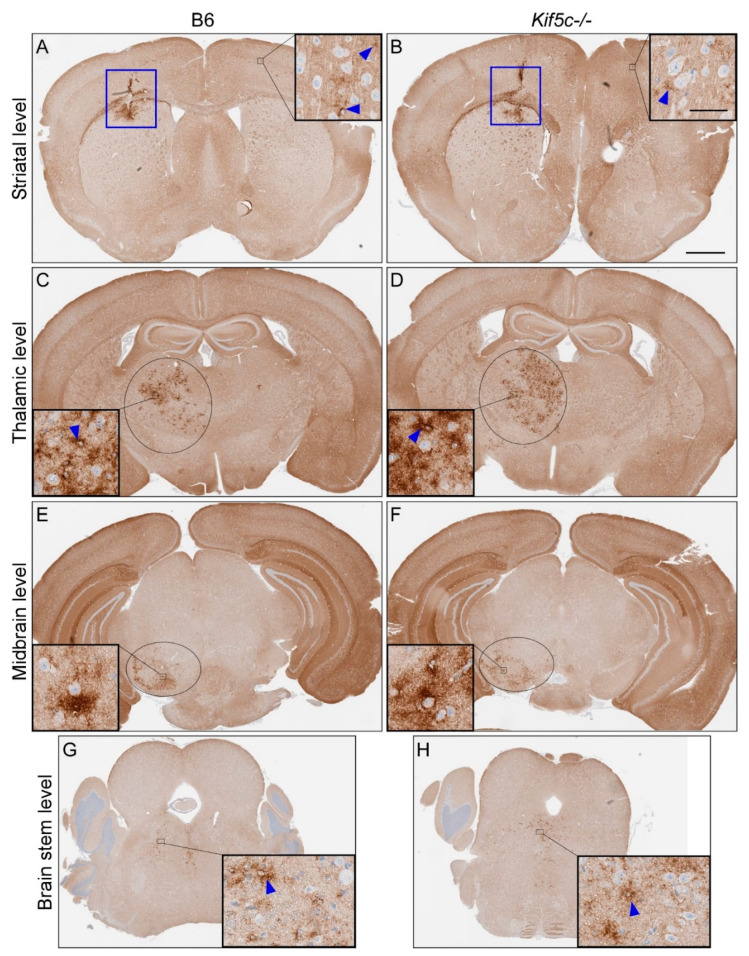
PrPSc staining of mouse brains 40 days post 22 L scrapie inoculation. IHC using anti-PrP antibody D13 was performed on coronal sections from four areas. A representative B6 mouse is shown on the left side, and a *Kif5c^−/−^* mouse on the right. (**A**,**B**) Coronal sections including the striatum. The nt is depicted with a blue box. The insets show higher magnification of the cerebral cortex with subtle PrPSc staining (blue arrowheads). (**C**,**D**) Coronal sections through the thalamus. The black oval depicts the approximate borders of the thalamus and the high magnification insets show focal PrPSc staining. (**E**,**F**) Midbrain sections including the substantia nigra (black ovals). Insets show higher magnification and PrPSc present in the substantia nigra. (**G**,**H**) Rostral pons sections and higher magnification insets showing PrPSc. The scale bar in B is 1 mm and applies to all the large panels. The scale bar in the inset within panel B is 50 µm and applies to all the insets.

We completed similar analysis of 22 L PrPSc deposition in four coronal brain sections, including the regions described above, from 3–5 prion-infected mice per mouse strain at 25, 40 and 60 dpi (Table 1). At 25 dpi, infection around the nt was clearly evident, but there was no evidence of PrPSc spread to the contralateral side of the brain at the same level in either B6 or *Kif5c^−/−^* mice (Figure 3A,B). However, subtle PrPSc deposits were observed at the level of the thalamus, substantia nigra and locus coeruleus region of the pons in at least some mice from each experimental group (Table 1 and Figure 3). At 25 dpi, all the *Kif5c^−/−^* mice had detectable PrPSc in the midbrain section containing the substantia nigra, but only 3 of 5 B6 mice were positive. In the brainstem, the majority of mice from both strains were positive for PrPSc deposition (Table 1). In some instances, mice were positive in the locus coeruleus but not in the substantia nigra. We believe this is likely due to the limited sensitivity of our IHC assay, and that the midbrain in these mice contains PrPSc but at a level below detection. When the ratio of positive and negative mice differed between mouse strains for a specific brain region and time point, we tested the significance using Fisher’s exact test. No significant differences in the spread of PrPSc were observed between B6 and *Kif5c^−/−^* mice at 25 dpi (Table 1). The extent of infection at each brain region also appeared equivalent between B6 and *Kif5c^−/−^* mice based on subjective scoring of PrPSc levels present (Table 1 and Figure 3). At 40 dpi (Table 1 and Figure 4), and 60 dpi (Table 1), the levels of PrPSc deposition had increased substantially in all the sections examined in both B6 and *Kif5c^−/−^* mice. PrPSc staining was also first detected in the contralateral cerebral cortex of both B6 and *Kif5c^−/−^* mice at 40 dpi (Figure 4A,B).

### 3.2. Stereotactic Kinetics of ME7 Scrapie at 40 Days Post-Inoculation (dpi)

**Figure 5:**
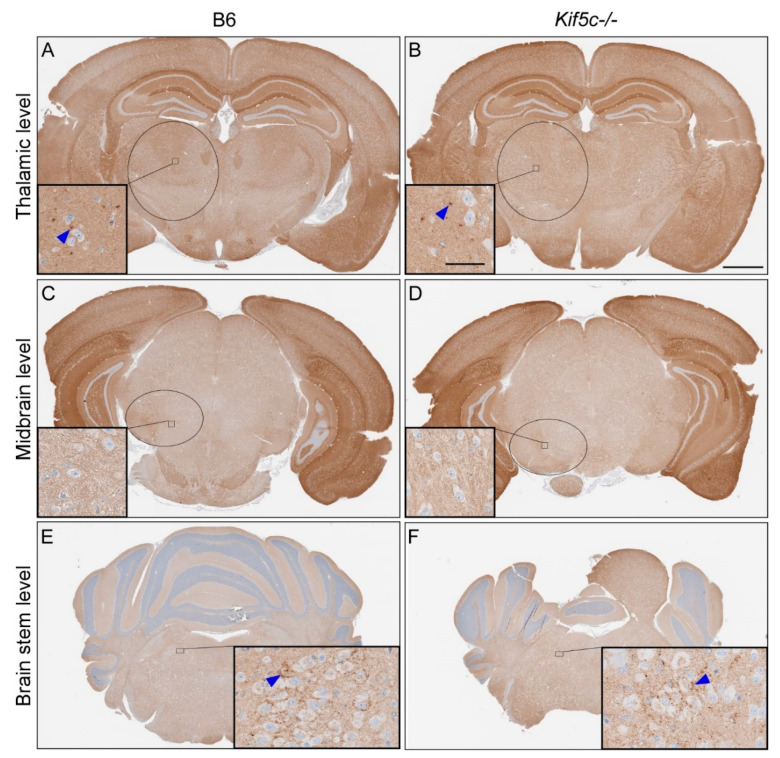
PrPSc staining of mouse brains 40 days post ME7 scrapie inoculation. IHC using anti-PrP antibody D13 was performed on coronal sections from three areas. A representative B6 mouse is shown on the left side, and a *Kif5c^−/−^* mouse on the right. (**A**,**B**) Coronal sections through the thalamus. The black oval depicts the approximate borders of the thalamus and the high magnification insets show very subtle, focal PrPSc staining (blue arrowheads). (**C**,**D**) Midbrain sections including the substantia nigra (black ovals). Insets show higher magnification, no PrPSc was observed in the mice shown. (**E**,**F**) Rostral pons sections and higher magnification insets showing perineuronal PrPSc. The scale bar in B is 1 mm and applies to all the large panels. The scale bar in the inset within panel B is 50 µm and applies to all the insets.

Next, we tested whether the findings with 22 L would be mirrored by ME7, a strain of mouse adapted scrapie that is more closely associated with neurons than 22 L [19]. Previously, we have reported that the early disease tempo of ME7 was much slower than 22 L and ME7 PrPSc was barely detectable in thalamus at 40 dpi [19]. Since comparisons of spreading tempo would be most accurate when PrPSc is near the limit of detection, we compared stereotactically inoculated *Kif5c^−/−^* and B6 mice at 40 dpi. Mice were euthanized and coronal sections through the thalamus, substantia nigra and pons were analyzed by IHC for PrPSc using anti-PrP antibody D13. At the level of the thalamus, subtle PrPSc deposits were present in both *Kif5c^−/−^* and B6 mice (Figure 5A,B). The coronal sections that included the substantia nigra were negative in four B6 mice whereas three of five *Kif5c^−/−^* mice had detectable PrPSc in the substantia nigra, however the differences were not statistically significant (*p* = 0.17; Table 1). More caudally in the brain, perineuronal PrPSc deposition was observed in the rostral pons of about half of all the mice tested from both groups (Figure 5E,F and Table 1). Collectively the ME7 infection data suggested no difference in early spreading kinetics between *Kif5c^−/−^* and B6 mice.

### 3.3. 22 L Scrapie-Infected Kif5c^−/−^ and B6 Mouse Survival Curve and Neuropathology

**Figure 6:**
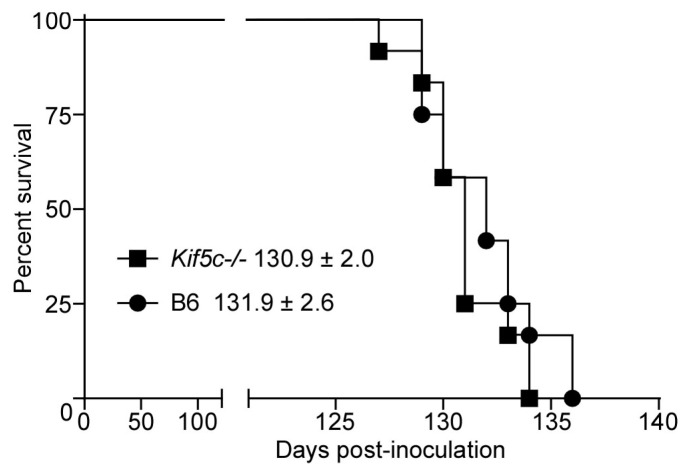
Survival curve of B6 and *Kif5c^−/−^* mice following 22 L scrapie infection. Twelve mice of each strain were inoculated stereotactically in the striatum with 0.5 µL of 10% 22 L scrapie brain homogenate. Mice were euthanized when advanced signs of terminal disease were present. Black squares indicate *Kif5c^−/−^* mice, black circles indicate B6 mice. The mean ± standard deviation (SD) for each strain are shown adjacent to the legend. Statistical analysis of the survival curve using the Log-rank (Mantel–Cox) test indicated no difference (*p* = 0.3152).

**Figure 7:**
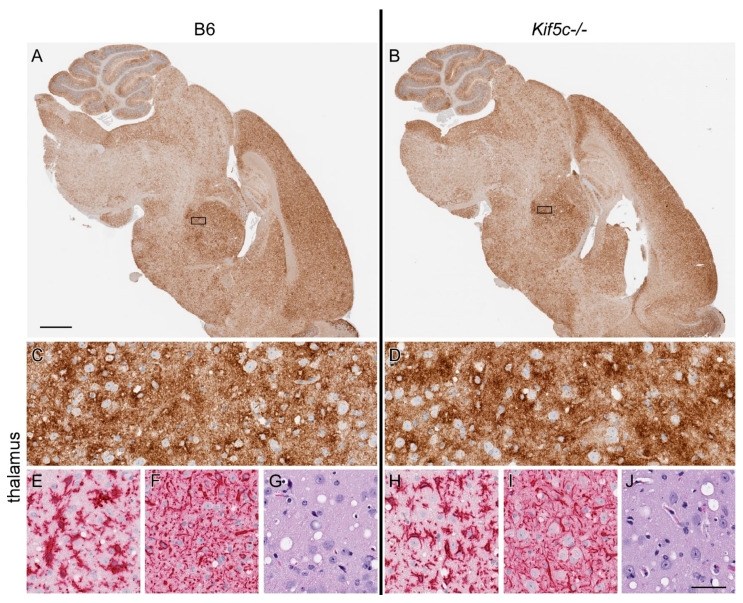
PrPSc deposition and neuropathology in brain collected from terminally sick 22 L-infected B6 and *Kif5c^−/−^* mice at 129 dpi. A representative B6 mouse is shown on the left side, and a *Kif5c^−/−^* mouse on the right. (**A**,**B**) IHC using anti-PrP antibody D13 (brown) performed on sagittal brain sections. PrPSc is widely distributed in both strains of mice. The black rectangle indicates the approximate area of thalamus shown at higher magnification in panels C–J. (**C**,**D**) High magnification D13 IHC. (**E**,**H**) Anti-Iba1 IHC showing activated microglia in thalamus. (**F**,**I**) Anti-GFAP IHC showing astrocytosis. (**G**,**J**) Hematoxylin and eosin (H&E) staining demonstrating spongiform lesions. No differences were observed in the distribution or amount of PrPSc, gliosis or spongiform degeneration between the two mouse strains. The scale bar in A is 1 mm and applies to panels A and B. The scale bar in J is 50 µm and applies to panels C–J.

We infected *Kif5c^−/−^* and B6 mice with 22 L scrapie and allowed them to reach terminal disease to determine whether absence of the KIF5C motor could alter survival times, neuropathology or the distribution of PrPSc in brain at the end stage of disease. Mice were infected using the same targeted inoculation method into the striatum with a small volume of concentrated 22 L to minimize artificial spread or inconsistency in inoculation location. Following inoculation, mice were observed by personnel blinded to the mouse genotype for onset of clinical signs and mice were euthanized when they developed consistent, advanced clinical signs. No differences were observed in survival times of B6 or *Kif5c^−/−^* mice (Figure 6). IHC for PrPSc distribution (anti-PrP antibody D13), microgliosis (anti-Iba1), astrogliosis (anti-GFAP) and neuropathology (H&E) was performed on five brains per experimental group. No differences in PrPSc amount or distribution were found (Figure 7A–D), nor was the level of gliosis altered between terminal groups (Figure 7E,F,H,I). Spongiform degeneration severity and distribution also appeared consistent between *Kif5c^−/−^* and B6 mice (Figure 7G,J).

## 4. Discussion

Scrapie prions are known to follow neuronal pathways from sites of injection or ingestion to various target areas in the nervous system [25]. In our experiments we tested whether deletion of the molecular motor, KIF5C, would alter the tempo of scrapie spread and pathogenesis. KIF5C was selected for our study since it had been previously identified as one of the primary anterograde motors involved in the transport of PrPC vesicles in axons of hippocampal neurons [17]. Our targeted, stereotactic inoculation method provided a consistent and reproducible infection model to closely follow prion spread at early time points post-inoculation and allowed for relevant survival time comparisons. Our results showed that the absence of KIF5C in *Kif5c^−/−^* mice did not result in delayed long-distance spread of PrPSc in either 22 L or ME7 mouse adapted scrapie infections. While our data do not show that the tempo of PrPSc infection is altered in B6 versus *Kif5c^−/−^* mice within our experimental paradigm, some caveats to our design are important to note.

First, the distribution of KIF5C in brain is not uniform, as this motor has been shown to be differentially expressed throughout the nervous system in subsets of neurons [26] and the Allen Mouse Brain Atlas 2004 (http://mouse.brain-map.org/experiment/show/74511818 (accessed on 19 May 2021)). Thus, it is possible that differences in axonal transport via KIF5C might be revealed in localized areas of the brain and more focused inoculation experiments would be necessary to address this possibility. Second, it is possible that kinesin motors other than KIF5C are involved in PrP transport. These could include kinesin-1B (KIF5B), which has also been implicated in PrPC vesicle movement [17]. Thus, this kinesin-1 motor redundancy could mask differences in PrPSc spread. Third, the PrPSc might spread via interstitial brain fluid (ISF), CSF, blood or lymph. These types of spread are usually slower than the spread seen in typical prion diseases and do not follow neuro-anatomical pathways [25]. However, if these pathways contribute to PrPSc spread, it is conceivable that a putative role of kinesin-1 in the transport of PrPSc within axons could be obscured by these other routes. Fourth, PrPSc has also been postulated to spread along neurons by a domino effect involving repeated conversion of membrane-anchored PrP [27,28]. Moreover, during conversion, small oligomeric PrPSc are created [29,30], and these could spread by kinesin-mediated axonal transport but go undetected with the resolution used in our assays. Fifth, lysosomes, exosomes, tunneling nanotubes, microsomes and extracellular vesicles have also been hypothesized as routes of spread for PrPSc [1,31]. A final consideration to explain our equivalent observation of early PrPSc deposition in the thalamus, substantia nigra and pons is the fact that the neuronal interconnectivity in the brain is extremely complex, with abundant crosstalk. In addition to receiving input from the striatum, the intralaminar nuclei of the thalamus, substantia nigra pars compacta and pedunculopontine nucleus of the pons also project neurons to the striatum, making it possible that retrograde transport in these neurons could have transported the PrPSc seen in our study. More definitive experiments need to be conducted to conclude whether neuronal versus non-neuronal pathways are responsible for the net spread of PrPSc.

Three other groups have tested whether alterations in axonal transport mechanisms can alter scrapie incubation periods. Hafezparast et al. studied mouse scrapie infection in a *Loa* mouse that carries a point mutation in the heavy chain subunit of cytoplasmic dynein resulting in impaired fast retrograde transport in spinal cord motor neurons [32]. *Loa* mice infected with scrapie strain RML by either peripheral or intracerebral routes did not differ in scrapie incubation periods from non-mutant control mice. Their results showed that dynein, a molecular motor responsible for retrograde transport, was not critical for spread of scrapie infection in peripheral nerves. Kunzi et al. took a more global approach to axonal transport by studying RML scrapie prion infection in mice that overexpressed 4-repeat human tau [33]. The tau-overexpressing mice had impaired anterograde and retrograde fast axonal transport, although the mechanism causing the impairment was not fully understood. Following inoculation of the sciatic nerve, no differences were seen in scrapie incubation periods compared to non-mutant controls. Heisler et al. identified muskelin as a key adapter protein that could complex with either dynein or KIF5C and coordinate directional transport [18]. Depletion of muskelin altered PrPC vesicle transportation resulting in less PrPC degradation by lysosomes and an increase in PrPC transport to the plasma membrane. When muskelin-deficient mice were infected with RML scrapie, the mice succumbed to disease much faster, likely due to decreased PrPSc degradation and increased vesicle delivery to the plasma membrane. While these data do not directly implicate KIF5C as a transporter of PrPSc, the indirect evidence that PrPSc is likely also transported by molecular motors is compelling. However, the Hafezparast and Kunzi studies and our current work suggest that alterations in prion spread and pathogenesis are not detectable when kinesin and dynein are mutated or deleted in mice.

The failure to slow the tempo of prion spread in our current experiments and those of others might merely reflect the lack of detailed knowledge of the possible transport mechanisms available for prion aggregates in these rather crude in vivo models. Alternatively, PrPSc may use molecular motors to move within the axon, but the rate-limiting step for overall PrPSc pathogenesis could occur at a different location, such as the synapse, where an entirely different mechanism would be required for spread and uptake of PrPSc.

## Figures and Tables

**Table 1:** Early spread of infectious prions (PrPSc) in brains of C57BL/6 and *Kif5c^−/−^* mice following stereotactic inoculation.

Scrapie Strain	Mouse Strain	Time Point (dpi ^2^)	Brain Region									
			Striatum (0) ^1^		Thalamus (3.2) ^1^		Midbrain (5) ^1^			Rostral Pons (7) ^1^		
			PrPSc Present ^3^	PrPSc Score ^4^	PrPSc Present ^3^	PrPSc Score ^4^	PrPSc Present ^3^	PrPSc Score ^4^	*p*-Value ^5^	PrPSc Present ^3^	PrPSc Score ^4^	*p*-Value ^5^
22 L	B6	7	2/2	1	0/2	0	0/2	0		0/2	0	
	*Kif5c^−/−^*	7	2/2	1	0/2	0	0/2	0		0/2	0	
	B6	25	5/5	2	5/5	1	3/5	0–1	0.46	4/5	0–1	>0.99
	*Kif5c^−/−^*	25	3/3	2	3/3	1	3/3	1		2/3	0–1	
	B6	40	4/4	3	4/4	2	4/4	2		4/4	1	>0.99
	*Kif5c^−/−^*	40	4/4	3	4/4	2	4/4	2		3/4	1	
	B6	60	3/3	2–3	3/3	3	3/3	1–2		3/3	1–2	
	*Kif5c^−/−^*	60	3/3	3	3/3	3	3/3	2		3/3	1–2	
ME7	B6	40	Not tested		4/4	1	0/4	0	0.17	2/4	0–1	>0.99
	*Kif5c^−/−^*	40	Not tested		5/5	1	3/5	0–1		3/5	0–1	

^1^ The approximate distance (mm) from the needle track is provided in parenthesis; ^2^ dpi: days post-inoculation; ^3^ Following stereotactic inoculation of scrapie into the striatum, mice were euthanized at different time points and brains were collected and analyzed for PrPSc deposition using immunohistochemical (IHC) methods. For each mouse, four different brain regions were analyzed for PrPSc deposition. The numerator shows the number of mice positive for the specific brain region, the denominator shows the number of mice tested; ^4^ PrPSc deposition intensity was given a subjective score from 0–4 with 0 being negative and 4 being a strong positive (see methods for additional detail); ^5^ *p*-values were calculated using Fisher’s exact test when the ratio of positive and negative mice differed between mouse strains at a specific time point and brain region. No significant differences were observed between B6 and *Kif5c^−/−^* mice.

## Data Availability

Data available upon request.
